# Enhancement of the priming efficacy of DNA vaccines encoding dendritic cell-targeted antigens by synergistic toll-like receptor ligands

**DOI:** 10.1186/1471-2172-10-43

**Published:** 2009-08-03

**Authors:** Claudius Grossmann, Matthias Tenbusch, Godwin Nchinda, Vladimir Temchura, Ghulam Nabi, Geoffrey W Stone, Richard S Kornbluth, Klaus Überla

**Affiliations:** 1 Department of Molecular and Medical Virology, Ruhr-Universität Bochum, Universitätsstraße 150, 44801 Bochum, Germany; 2 Laboratory of Cellular Physiology and Immunology, The Rockefeller University, 1230 York Avenue, New York, NY 10021, USA; 3 Department of Microbiology and Immunology, Miller School of Medicine, University of Miami, 1580 NW 10th Avenue (R138), Miami, FL 33136, USA; 4 Department of Medicine, University of California, San Diego, 9500 Gilman Dr., La Jolla, California 92093-0679, USA

## Abstract

**Background:**

Targeting of protein antigens to dendritic cells (DC) via the DEC205 receptor enhances presentation of antigen-derived peptides on MHC-I and MHC-II molecules and, in the presence of costimulatory signals, antigen-specific immune responses. The immunogenicity and efficacy of DNA vaccination can also be enhanced by fusing the encoded antigen to single chain antibodies directed against DEC205. To further improve this strategy, we evaluated different toll-like receptor ligands (TLR) and CD40 ligands (CD40L) as adjuvants for DNA vaccines encoding a DEC205-single-chain antibody fused to the ovalbumin model antigen or HIV-1 Gag and assessed the priming efficacy of DNA in a DNA prime adenoviral vector boost immunization regimen.

**Results:**

Mice were primed with the adjuvanted DEC-205 targeted DNA vaccines and boosted with adenoviral vectors encoding the same antigens. CD8^+ ^T cell responses were determined after the adenoviral booster immunization, to determine how well the different DNA immunization regimens prime for the adenoviral boost. In the absence of adjuvants, targeting of DNA-encoded ovalbumin to DCs suppressed CD8^+ ^T-cell responses after the adenoviral booster immunization. CD8^+ ^T-cell responses to the DEC205 targeted DNA vaccines increased only slightly by adding either the TLR-9 ligand CpG, the TLR-3 ligand Poly I:C, or CD40 ligand expression plasmids. However, the combination of both TLR-ligands led to a strong enhancement of CD8^+ ^T-cell responses compared to a non-targeted DNA vaccine. This finding was confirmed using HIV Gag as antigen.

**Conclusion:**

Although DNA prime adenoviral vector boost immunizations belong to the strongest inducers of cytotoxic T cell responses in different animal models and humans, the CD8^+ ^T cell responses can be further improved by targeting the DNA encoded antigen to DEC205 in the presence of synergistic TLR ligands CpG and Poly I:C.

## Background

Depending on their state of maturation or activation dendritic cells (DC) can prime antigen-specific T cells with substantially different functional activities. While DC can mediate peripheral T cell tolerance in the steady-state, activation and maturation turns them into potent inducers of CD4^+ ^and CD8^+ ^T cell immunity (reviewed in [1]). Thus, targeting antigens to DCs is a promising strategy to improve prevention and treatment of autoimmunity and to raise the efficacy of vaccines against tumors and infectious diseases. For targeting of DC in vivo, most of the studies use recombinant protein antigens coupled or fused to an antibody specific for a DC surface marker. In addition to targeting the DCs their differentiation and activation status needs to be controlled in order to obtain the desired T cell response. A striking example of how DC activation and differentiation signals can modulate T cell responses has been obtained in experiments using an antibody to the DEC205 receptor (α-DEC) to target proteins coupled to the antibody to DCs [2]. Targeting of antigens to dendritic cells via the DEC205 receptor enhances presentation of antigen-derived peptides on MHC-I and MHC-II molecules [2,3]. Efficient loading of MHC-II molecules with DEC205-targeted antigenic peptides might occur during the recycling of the DEC205 receptor with its ligands through late, MHC-II-rich endosomal compartments [4]. MHC-I-restricted presentation of exogenous DEC-205-targeted antigens by dendritic cells in vivo was shown to be dependent on the transporter of antigenic peptides [3]. In the absence of costimulatory signals DEC205-targeted antibody fused to antigens induced initial T cell proliferation followed by peripheral deletion and unresponsiveness of CD4^+ ^and CD8^+ ^T cells [2,3]. Induction of regulatory T cells [2,3,5,6] and a novel form of peripheral tolerance [7] could also be observed after immunization with different DEC-205-targeted antigens in the absence of co-stimulation. Although the precise mechanisms leading to these different forms of peripheral tolerance remain to be defined, simultaneous co-stimulation via anti-CD40 antibodies and/or toll-like receptor (TLR) ligands induces antigen-specific CD4^+ ^and CD8^+ ^T cell immunity rather than tolerance [8-10].

Since gene-based vaccines are usually more potent inducers of cytotoxic T-cell responses than protein vaccines, we previously investigated whether the immunogenicity of DNA vaccines could be enhanced by targeting the encoded protein to DEC205 expressing dendritic cells [11]. In addition to the differences in immune responses induced by DNA and protein vaccines, a practical advantage of DNA vaccines encoding DC-targeted antigens is that the DNA vaccine can be produced in large scale under standardized conditions more or less independent of the vaccine antigen, while production and purification of recombinant protein vaccines needs to be adjusted for each antigen. Immunizing mice by *in vivo *electroporation with DNA vaccines encoding HIV Gag fused to single-chain antibodies to DEC205 allowed to reduce the DNA dose approximately 100-fold without impairment of T cell immunity and protection from viral challenge even in the absence of exogenous co-stimulation. Although this might allow overcoming the requirement for large doses, a key obstacle to the application of DNA vaccines in humans, the DC-targeted DNA vaccines did not enhance immune responses above the levels obtained by DNA immunization with high doses of non-targeted DNA vaccines.

Since the immunogenicity of viral vector vaccines can be improved by priming with DNA vaccines in various animal models including non-human primates (reviewed in [12]) and humans [13-15], we now explored whether targeting of DNA vaccine encoded antigens to DCs can increase the priming efficacy of the DNA vaccines in DNA prime viral vector boost regimens. In addition, the requirement for additional stimuli during priming with the DEC205-targeted DNA vaccines was analysed.

## Methods

### DNA and viral vector vaccines

Plasmids and adenoviral vectors expressing Ovalbumin or HIV GagPol, which were used in this study, are listed in Table 1. To target the plasmid encoded antigens to dendritic cells, they were fused to a single chain antibody directed against the DC surface receptor DEC205 as described previously. Plasmids encoding the antigens fused to a nonreactive antibody with different variable light- and heavy-chain V regions were used as controls [11]. Constructs were confirmed by sequence analysis. For *in vivo *experiments all plasmids were prepared using the Qiagen Endo-Free plasmid kit (Hilden, Germany). The Limulus Amebocyte Lysate QCL-1000^® ^kit (Cambrex, Charles City, IA, USA) was used to confirm that the Endotoxin concentration were below 0,1 EU (Endotoxin Units) per dose. Construction of the Ad-Ova, Ad-Hgp^syn^, and Ad-GFP vector has been previously described [16-18]. Vector particles grown on 293A cells were purified by the AdenoPack100 kit (Vivascience, Goettingen, Germany). Particle concentrations were determined by measuring the optical density and the 50% tissue culture infectious dose (TCID_50_) was determined on 293A cells. Mock immunizations were performed with an empty vector plasmid (pcDNA3.1+)

**Table 1:** Plasmids and adenoviral vectors

Construct	Backbone	Single chain antibody	Antigen	Ref.
DEC-OVA	pcDNA.3.1	Anti-DEC205	Ovalbumin	11
Con-OVA	pcDNA.3.1	Non-reactive	Ovalbumin	11
DEC-p41	pcDNA.3.1	Anti-DEC205	HIV Gag-p41	11
Con-p41	pcDNA.3.1	Non-reactive	HIV Gag-p41	11
Ad-OVA	AdV	---	Ovalbumin	16
Ad-Hgp^syn^	AdV	---	HIV GagPol	17
Ad-GFP	AdV	---	Green Fluorescence Protein	18

To study the adjuvant properties of multimerized CD40L we used plasmids encoding different multimerized forms of CD40L, which have been previously described [19]. CpG-C (Coley Pharmaceuticals, Wellesley, MA, USA) and Poly I:C (Invivogen, Toulouse, France) were used at a dose of 50 μg per injection.

### Immunization

6–8 week old female BALB/c and C57/Bl6 were obtained from Charles River (Sulzfeld, Germany) and Janvier (Le Genest-ST-Isle, France), respectively and housed in singly-ventilated cages in accordance with the national law and institutional guidelines. DNA vaccines (2–50 μg) were delivered subcutaneously into both anterior foot pads in a total volume of 100 μl PBS. Plasmids encoding multimerized forms of CD40L (50 μg) or the TLR Ligands CpG-C (Coley Pharmaceuticals, Wellesley, MA, USA) and Poly I:C (Invivogen, Toulouse, France) were mixed with the antigen expressing plasmids prior to injection in a final volume of 100 μl. CpG-C and Poly I:C were used at a dose of 50 μg per injection. Five weeks later mice were boosted with 5 × 10^8 ^adenoviral vector particles in 100 μl PBS by the same route.

### Tetramer staining

Ova-specific CD8^+ ^T-cell responses were measured seven days after the booster immunization by tetramer staining. After red blood cell lysis, 1 × 10^6 ^splenocytes were plated in 96-well round-bottom plates (Nunc, Wiesbaden, Germany) for each staining.

Cells were washed once in PBS/BSA/Azide and then incubated with 2 μl of SIINFEKL/H-2K^b^-APC tetramers (Sanquin, Amsterdam, NL) in total volume of 100 μl PBS/BSA/Azide for 40 min at room temperature. Afterwards surface staining with α-CD8-FITC was performed for 20 min at room temperature and cells incubated with 7-amino-actinomycin D (7-AAD) for 5 min to exclude dead cells from subsequent FACS analyses. Analysis was performed using a FACScalibur™ (BD Biosciences, Heidelberg, Germany).

### Intracellular cytokine staining (ICS)

To analyse antigen-specific T-cell responses via ICS splenocytes were stimulated for 6 h in the presence of 2 μM Monensin, which inhibits the cytokine secretion, and 1 μl α-CD107a-FITC, which is a marker for lymphocyte degranulation [20]. Cells were either stimulated by SIINFEKL (Ovalbumin_257–264_, 2 μg/ml) or AMQMLKETI (HIV Gag_197–205_, 2 μg/ml) and compared to non-stimulated cultures. After stimulation, surface staining was carried out with αCD8-PerCP or αCD4-FITC (BD Bioscience). Cells were fixed in 2% paraformaldehyde, followed by permeabilisation with 0,5% Saponin in PBS/BSA/Azide buffer. Cytokines were detected with αIFN-γ-PE and αIL-2-AlexaFluor647 (BD Bioscience).

### In vivo cytotoxicity assay

To analyse *in vivo *CTL responses, spleen cells were isolated from syngenic donor mice. Splenocytes were divided into two groups and labelled either with 6 μM (CFSE^high^) or 0.3 μM (CFSE^low^). After several washing steps the CFSE^high ^fraction was loaded for 30 min at 37°C with 10 μg/ml SIINFEKL (Ovalbumin_257–264_) or AMQMLKETI (HIV Gag_197–205_,) peptides. Peptide-loaded cells (CFSE^high^) were mixed in a one to one ratio with unloaded cells (CFSE^low^) and appropriately 1.5 × 10^7 ^cells in a total volume of 300 μl PBS were injected into the tail vein of immunized or control mice. One day post-injection mice were sacrificed. Spleen cells were isolated and the ratio of CFSE^high ^to CFSE^low ^labelled spleen cells were determined by flow cytometry.

### Statistical analysis

Statistical analysis was performed using the GraphPad Prism 4.0 software (Graph, software Inc., San Diego, CA, USA). In case the one-way ANOVA test revealed a statistical significant difference, the Bonferroni Multiple Comparison test was used to determine the level statistical significance between two groups.

## Results

In several experimental systems, DNA prime viral vector boost immunization regimens are the most potent inducers of CD8^+ ^T-cell responses (reviewed in [21]). Although the DNA prime itself only induces weak immune responses, CD8^+ ^T-cell responses are substantially higher after the viral boost compared to DNA prime DNA boost immunization or viral vector immunizations in the absence of DNA priming. We therefore modified the DNA priming conditions and analysed the effect of DNA priming on CD8^+ ^T-cell responses after a constant viral vector boost to directly assess the potency of the combined DNA prime viral vector boost regimen.

**Figure 1:**
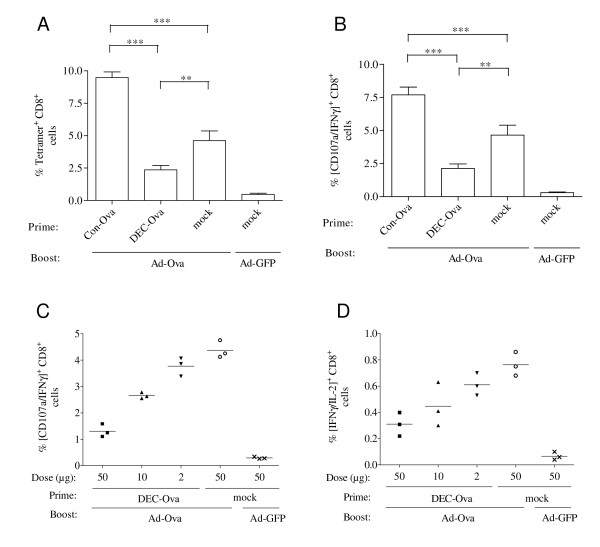
**CD8^+ ^T cell responses after priming with a DNA vaccine encoding DEC-205 targeted ovalbumin and boosting with an adenoviral vector**. Mice were immunized with the indicated plasmids prior to boosting with adenoviral vectors encoding ovalbumin (Ad-Ova) or GFP (Ad-GFP). Unless stated otherwise, a DNA dose of 50 μg was used. One week after the boost, the percentage of SIINFEKL/H-2K^b ^tetramer positive cells (A), IFN-γ and CD107a (B, C) or IFN-γ and IL-2 (D) double-positive cells after stimulation with the SIINFEKL peptide were determined. A, B) Mean percentages with SEM of three independent experiments with a total of eight mice/group are shown. Significant differences (Bonferroni Multiple Comparison test) between groups primed with different DNA vaccines and boosted with Ad-OVA are marked (* p < 0.05; ** p < 0.01; *** p < 0.001). C, D) Dose response curve for DEC-Ova priming. The percentage of IFN-γ and CD107a positive (C) and IFN-γ and IL-2 positive (D) cells of CD8^+ ^lymphocytes is shown for individual mice.

To determine the effect of targeting the antigen expressed by the DNA vaccine to DCs on the immunogenicity of a DNA prime viral vector boost regimen, C57/Bl6 mice were primed by subcutaneous immunization with a DNA vaccine encoding a DEC-205-single chain antibody fused to ovalbumin (DEC-OVA) and boosted with an adenoviral vector expressing ovalbumin (Ad-Ova). The Ad-Ova vector was used at a suboptimal dose (data not shown) of 5 × 10^8 ^particles corresponding to approximately 2 × 10^7 ^transducing units in order to better detect the influence of the DNA prime. Priming with the DEC205-targeted DNA vaccine (pDEC-Ova) reduced the percentage of Ova tetramer-positive CD8^+ ^T-cells after the Ad-Ova boost in comparison to a non-targeted DNA vaccine (pCon-Ova) encoding ovalbumin fused to a non-reactive single-chain antibody (Figure 1A). The functional activity of OVA-specific CD8^+ ^T cells was also assessed by stimulation with the SIINFEKL peptide followed by staining for interferon-γ and the degranulation marker CD107a. Consistent with the tetramer staining, the percentage of Ova-specific CD107a- and IFNγ-positive CD8^+ ^T cells was lower after priming with the DEC205-targeted DNA vaccine (Figure 1B). The CD8^+ ^T cell response in mice primed with the DEC205-targeted DNA Ova vaccine was even lower than in mice that received the Ad-Ova vaccine in the absence of a DNA Ova prime, indicating that DEC-205 targeting during DNA priming suppressed CD8^+ ^T cell responses induced by the Ad-Ova vector. This suppressive effect was dependent on the dose of the DEC205-targeted DNA vaccine (Figure 1C) and was also observed for the subpopulation of CD8+ T cells co-expressing IFN-γ and interleukin-2 (Figure 1D). Reduction of this subpopulation is particularly problematic, since secretion of IL-2 can promote the expansion of T cells and has been shown to enhance CD8+ T-cell memory function [22].

**Figure 2:**
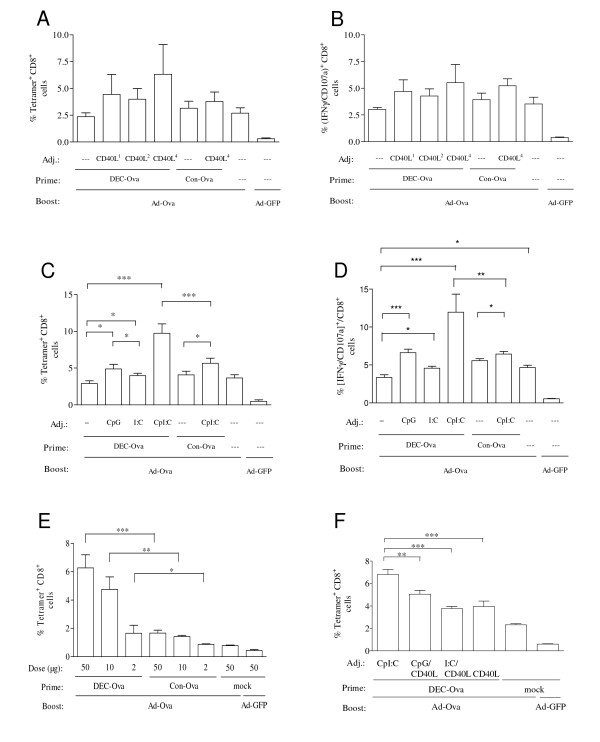
**CD8^+ ^T-cell responses after priming with DEC-205 targeted DNA vaccines in the presence of costimulatory adjuvants and boosting with an adenoviral vector**. Mice were primed with DEC-Ova or Con-Ova in the presence or absence of the indicated adjuvants (Adj.) prior to boosting with adenoviral vectors encoding ovalbumin (Ad-Ova) or GFP (Ad-GFP). The percentage of SIINFEKL/H-2K^b ^tetramer positive cells (A, C, E, F) and IFN-γ and CD107a double-positive cells after stimulation with the SIINFEKL peptide (B, D) were determined after the boost. Unless stated otherwise, DEC-Ova and Con-Ova were used at a dose of 50 μg. A, B, F) Fifty μg of plasmids encoding a soluble trimer (CD40L^1^), a dimeric form (CD40L^2^) or a tetrameric form of that trimer (CD40L^4^) were co-administered with the DNA vaccine. C, D, F) CpG, Poly I:C or the combination of CpG and Poly I:C (CpI:C) were coinjected with the plasmids as indicated. E) CpI:C was added during DNA priming. Mean percentages with SEM of two independent experiments with a total of eight mice/group are shown (A-D). Mean percentages and SEM of one experiment with four (E) and six (F) mice per group are shown. For reason of clarity, the level of significance in the Bonferroni Multiple Comparison test is only indicated for selected group to group comparisons (* p < 0.05; ** p < 0.01; ***p < 0.001).

Since co-stimulation changes the outcome of DEC205-targeted protein immunizations from peripheral tolerance to immunity, the DEC205-targeted DNA vaccines were co-injected with expression plasmids for trimeric soluble CD40L, a dimeric form of this trimer, or a tetrameric form of the trimeric CD40L [19]. Although there might be a trend to enhancement of the priming efficacy of the DEC205-targeted DNA vaccine by co-expression of the tetrameric CD40L, this did not reach statistical significance (Figure 2A, B). We therefore also explored the effect of two toll-like receptor (TLR) ligands: CpG-C and Poly I:C. CpG-C binds to TLR 9 leading to signalling via the myeloid differentiation primary-response protein 88 (MyD88), while Poly I:C triggers TLR 3 resulting in the activation of TRIF (TIR domain-containing adaptor protein inducing interferon-β) (reviewed in [23]). In addition, Poly I:C also activates the melanoma differentiation-associated gene-5 (MDA-5) pattern recognition receptor [24], that is also expressed by many cell types and involved in anti-viral immune responses (reviewed in [25]).

Addition of CpG or Poly I:C during priming with the DEC205-targeted DNA led to a small but significant enhancement of CD8^+ ^T cell responses after the Ad-Ova boost. However, a much stronger stimulation of the CD8^+ ^T cell responses was observed with the combination of CpG and Poly I:C, designated CpI:C. Importantly, the DEC205-targeted DNA vaccine in the presence of CpI:C primed significantly stronger CD8^+ ^T cell responses than the adjuvanted non-targeted DNA vaccine (Figure 2C, D). This could be confirmed for a wide dose range of the DEC-205-targeted DNA vaccine (Figure 2E). Priming with 2 μg of the DEC205-targeted DNA vaccine primed for the viral vector boost as efficiently as 50 μg of non-targeted DNA vaccine. Co-stimulation with different combinations of the TLR ligands and the CD40L did not improve CD8^+ ^T cell responses of the DNA prime adenoviral vector boost immunization above the levels obtained by co-stimulation with CpI:C (Figure 2F).

**Figure 3:**
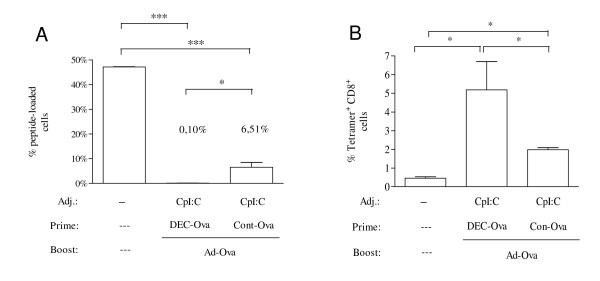
***In vivo *CTL activity after priming with adjuvanted DNA vaccine encoding ovalbumin and adenoviral vector booster immunization**. Mice (n = 3) were primed either with DEC-Ova or Con-Ova in the presence of CpI:C and boosted with the Ad-Ova vector. One week after the boost, mice received a 1 to 1 mixture of SIINFEKL peptide-loaded and non-loaded syngenic donor splenocytes also differing in the intensity of the CFSE-labelling. One day after immunization, the percentage of SIINFEKL loaded donor cells (A) and tetramer ^+ ^CD8^+ ^cells (B) was determined. The levels of significance in the Bonferroni Multiple Comparison test are indicated (* p < 0.05; ** p < 0.01; ***p < 0.001).

To determine whether the improvement of CD8^+ ^T cell responses as determined by *in vitro *assays would be of functional relevance *in vivo*, mice were primed with the DEC-205-targeted DNA vaccine or the non-targeted DNA vaccine in the presence of CpI:C and boosted with Ad-Ova. Subsequently, immunized mice were co-injected with differentially CFSE-labelled syngeneic spleen cells either loaded with an immunodominant Ova peptide or not loaded with exogenous peptide. One day after injection, the percentage of Ova-peptide-loaded cells among the transferred cells declined to hardly detectable levels in mice primed with the DEC205-targeted DNA vaccine in the presence of CpI:C (Figure 3A). The *in vivo *cytotoxic activity for Ova-peptide loaded cells was significantly lower in mice immunized with the non-targeted DNA prime Ad-Ova boost. As expected, the percentage of Ova-peptide loaded cells was inversely correlated to the Ova tetramer positive CD8^+ ^T cells (Figure 3B) measured in parallel for the same mice.

**Figure 4:**
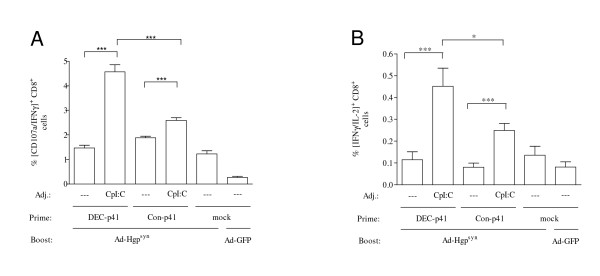
**HIV Gag-specific CD8^+ ^T cell responses**. Mice were primed with a DNA vaccine encoding DEC205-targeted HIV p41 Gag (DEC-p41) or control plasmids (Con-p41, mock) with or without CpI:C prior to boosting with an adenoviral vectors encoding HIV GagPol (Ad-Hgp^syn^) or GFP (Ad-GFP). One week after the boost, the percentage of CD8^+ ^cells also positive for IFN-γ and CD107a (A) or IFN-γ and IL2 (B) after stimulation with an immunodominant HIV Gag peptide were determined. Mean percentages with SEM of two independent experiments with a total of eight mice/group are shown. For reason of clarity, the level of significance in the Bonferroni Multiple Comparison test is only indicated for selected group to group comparisons (* p < 0.05; ** p < 0.01; ***p < 0.001).

The HIVp41 Gag antigen was used as an independent antigen to confirm the key findings we had obtained with the ovalbumin model antigen. Since the immunodominant Gag epitope, AMQMLKETI, is H2-Kb restricted, Balb/C mice were primed with a DNA vaccine encoding DEC205-targeted HIVp41 Gag in the presence or absence of CpI:C prior to a boost with an adenoviral vector encoding HIV GagPol (Ad-Hgp^syn^). Addition of CpI:C during priming with the DEC205-targeted HIV-p41 Gag DNA enhanced Gag-specific IFN-γ producing CD8^+ ^T cells approximately 3-fold (Figure 4A). T-cells producing multiple cytokines appear to be particularly indicative of protective immunity (reviewed in [26]). We therefore also analysed the influence of CpI:C on the capacity of CD8^+ ^T cells to simultaneously produce IFN-γ and IL2. Adding CpI:C during priming with the DEC205-targeted HIV-p41 Gag DNA enhanced the percentage of CD8^+ ^T cells double positive for IFN-γ and IL2 approximately 4-fold (Figure 4B). The CD8^+ ^T cell response in mice primed with the DEC205-targeted HIVp41 DNA in the presence of CpI:C was also higher than in mice primed with adjuvanted non-targeted DNA vaccines confirming the results obtained with the ovalbumin model antigen. However, the suppression of CD8^+ ^T cell responses by targeting the encoded ovalbumin to DEC205 in the absence of adjuvant could not be observed for the DEC205-targeted HIVp41 DNA vaccine.

**Figure 5:**
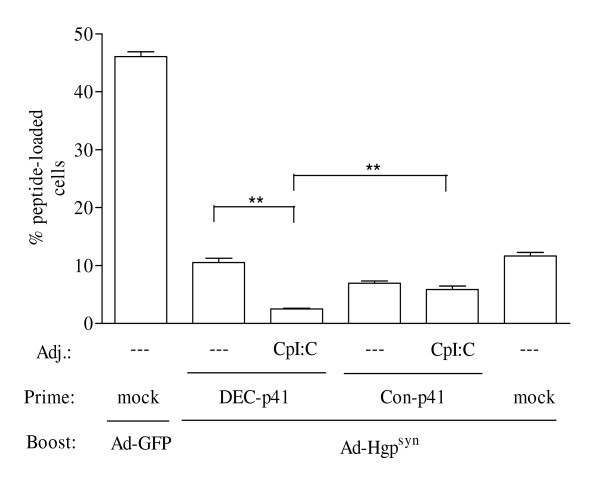
**HIV Gag-specific *in vivo *CTL activity**. Mice (n = 4) were immunized as described in Figure 4. One week after the boost, mice received a 1 to 1 mixture of HIV Gag peptide-loaded and non-loaded syngenic donor splenocytes also differing in the intensity of the CFSE-labelling. One day after immunization, the percentage of HIV Gag peptide-loaded donor cells was determined. For reason of clarity, the level of significance in the Bonferroni Multiple Comparison test is only indicated for selected group to group comparisons (** p < 0.01).

The CD8^+ ^T cell responses were further analysed by *in vivo *CTL assays. Priming with the DEC205-targeted HIVp41 DNA in the absence of CpI:C did not enhance *in vivo *cell killing after the adenoviral vector immunization, while a priming effect of the non-targeted DNA vaccine could be observed (Fig 5). Adding CpI:C to the non-targeted DNA vaccine slightly enhanced in vivo cell killing, but this difference did not reach statistical significance. The strongest *in vivo *CTL activity was obtained by addition of CpI:C during DEC205-targeted HIVp41 DNA priming confirming the results of the *in vivo *CTL assays with the Ova model antigen.

## Discussion

Adjuvanting DEC205-targeted DNA vaccines with TLR-ligands substantially enhanced CD8^+ ^T-cell responses of the DNA prime viral vector boost regimen. The DEC205-targeted DNA prime in the presence of CpG and Poly I:C adjuvants followed by an adenoviral vector boost induced stronger CD8^+ ^T cell responses than non-targeted DNA prime adenoviral vector boost immunizations. Since the latter regimen is considered to be one of the most efficient ways to induce cytotoxic T cell responses in rodents, non-human primates and humans this could be an important achievement. Although pre-existing immunity of human adenoviral vectors could reduce the immune responses induced, several strategies to overcome this limitation, such as the use of rare adenoviral serotypes (reviewed in [27]) and non-human adenoviruses [28], are presently persued.

The DEC205-targeted DNA prime adenoviral boost regimen also seems to induce substantially stronger CD8^+ ^T cell responses in comparison to previous DEC205-targeted protein vaccines and DEC205-targeted DNA immunization experiments [8,11]. In these previous experiments with DEC205-targeted protein vaccines co-stimulation with anti-CD40 antibody induced optimal CD8^+ ^and CD4^+ ^T-cell responses [8,10]. We therefore also explored whether co-expression of different forms of CD40L could increase the priming efficacy of DEC205-targeted DNA vaccines. These CD40L expression plasmids enhance the immunogenicity of DNA vaccines after repeated intramuscular co-delivery with the DNA vaccine [19]. We also observed a trend towards enhancement of CD8^+ ^T-cell responses by co-expression of CD40L during DNA priming via a single subcutaneous DNA immunization. However, due to a larger variation in immune responses using the subcutaneous vaccination route, the stimulatory effect by co-expression of CD40L did not reach statistical significance. Our results also indicate that DEC205-targeting of antigens encoded by DNA vaccines could be a double-edged sword. In the absence of additional stimuli, suppression of CD8^+ ^T cell responses was observed for the ovalbumin model antigen, but not for HIVp41 Gag. What governs this different outcome is not understood, but could be related to the affinity of the T-cell receptor for the antigenic peptide/MHC complex or antigen-mediated modulation of the state of differentiation or activation of the DCs. Similar mechanisms were proposed to underlay the different forms of peripheral tolerance induced by DEC205-targeted peptide vaccines [6].

At a first glance, the suppressive effects we observed in our non-adjuvanted ovalbumin DNA prime adenoviral boost regimen also seems to contradict the enhancement of DNA vaccination efficacy by DEC205-targeted HIVp41 Gag DNA in the absence of co-stimulation [11]. However, in the latter study, the DEC205-targeted DNA vaccine was administered by *in vivo *electroporation. Since *in vivo *electroporation induces a strong inflammatory response [29], the delivery mode might have overcome the requirement for an additional stimulus, which we observed in the present study after subcutaneous immunization with the DNA vaccines. The inflammatory response induced by electroporation might explain why the DEC205-targeted HIVp41 DNA vaccine enhanced Gag-specific immune responses in the absence of adjuvant after *in vivo *electroporation, but not after subcutaneous immunization.

The type of immune response induced by DEC205-targeted protein or DNA vaccines critically depends on the activation and maturation status of the targeted DCs. This may vary within one individual depending on the site of vaccine injection and/or local infections and between individuals due to differences in the overall activation status of the immune system. It might therefore be important to override the endogenous differentiation status of the DCs at the injection site of DEC205-targeted vaccines. For DEC205-targeted DNA vaccines, the innate stimulatory properties of the injected DNA itself does not seem to be sufficient for all antigens as indicated by the suppressive effects induced by the DEC205-targeted DNA expressing ovalbumin. Although the detection of antigen-specific, IFN-γ and interleukin 2 secreting CD8+ T cells after priming with DEC205-targeted DNA vaccines in the presence of co-stimuli is suggestive for induction of memory immune responses, it will also be important to evaluate memory immune responses directly, since they are necessary for long-term protective efficacy after vaccination.

## Conclusion

The present study demonstrates that antigen specific CD8^+ ^T cell responses induced by DNA prime adenoviral vector boost regimens can be consistently enhanced by priming with DEC205-targeted DNA vaccines in the presence of TLR 3 and 9 ligands. Simultaneous stimulation of the TRIF and MyD88 pathways via TLR3 and TLR9 has been shown previously to lead to DCs with enhanced T helper type 1 polarizing capacity [30-32]. Thus, combining adjuvants acting synergistically on DCs with DC-targeted vaccines seems to be an particularly attractive strategy for development of prophylactic or therapeutic vaccines against chronic viral infections such as HIV and HCV, in which strong cytotoxic T cell responses are considered to be of benefit.

## Authors' contributions

CG performed most of the immunization experiments, performed the statistical analysis, and participated in the drafting of the manuscript. MT constructed the adenoviral vector vaccines and established the CD8^+ ^T cell assays. GoN constructed the single-chain expression plasmid and participated in the design of the experiments. VT established and performed the in vivo CTL assays. GhN participated in the immunization experiments. GWS and RSK constructed the CD40L expression plasmids and participated in the interpretation of the data and the writing of the manuscript. KÜ conceived of the study, and participated in its design and coordination and drafted the manuscript. All authors read and approved the final version of the manuscript.

## References

[B1] Steinman RM, Banchereau J (2007). Taking dendritic cells into medicine. Nature.

[B2] Hawiger D, Inaba K, Dorsett Y, Guo M, Mahnke K, Rivera M, Ravetch JV, Steinman RM, Nussenzweig MC (2001). Dendritic cells induce peripheral T cell unresponsiveness under steady state conditions in vivo. J Exp Med.

[B3] Bonifaz L, Bonnyay D, Mahnke K, Rivera M, Nussenzweig MC, Steinman RM (2002). Efficient targeting of protein antigen to the dendritic cell receptor DEC-205 in the steady state leads to antigen presentation on major histocompatibility complex class I products and peripheral CD8+ T cell tolerance. J Exp Med.

[B4] Mahnke K, Guo M, Lee S, Sepulveda H, Swain SL, Nussenzweig M, Steinman RM (2000). The dendritic cell receptor for endocytosis, DEC-205, can recycle and enhance antigen presentation via major histocompatibility complex class II-positive lysosomal compartments. J Cell Biol.

[B5] Mahnke K, Qian Y, Knop J, Enk AH (2003). Induction of CD4+/CD25+ regulatory T cells by targeting of antigens to immature dendritic cells. Blood.

[B6] Kretschmer K, Apostolou I, Hawiger D, Khazaie K, Nussenzweig MC, von BH (2005). Inducing and expanding regulatory T cell populations by foreign antigen. Nat Immunol.

[B7] Hawiger D, Masilamani RF, Bettelli E, Kuchroo VK, Nussenzweig MC (2004). Immunological unresponsiveness characterized by increased expression of CD5 on peripheral T cells induced by dendritic cells in vivo. Immunity.

[B8] Bonifaz LC, Bonnyay DP, Charalambous A, Darguste DI, Fujii S, Soares H, Brimnes MK, Moltedo B, Moran TM, Steinman RM (2004). In Vivo Targeting of Antigens to Maturing Dendritic Cells via the DEC-205 Receptor Improves T Cell Vaccination. J Exp Med.

[B9] Trumpfheller C, Caskey M, Nchinda G, Longhi MP, Mizenina O, Huang Y, Schlesinger SJ, Colonna M, Steinman RM (2008). The microbial mimic poly IC induces durable and protective CD4+ T cell immunity together with a dendritic cell targeted vaccine. Proc Natl Acad Sci USA.

[B10] Trumpfheller C, Finke JS, Lopez CB, Moran TM, Moltedo B, Soares H, Huang Y, Schlesinger SJ, Park CG, Nussenzweig MC (2006). Intensified and protective CD4+ T cell immunity in mice with anti-dendritic cell HIV gag fusion antibody vaccine. J Exp Med.

[B11] Nchinda G, Kuroiwa J, Oks M, Trumpfheller C, Park CG, Huang Y, Hannaman D, Schlesinger SJ, Mizenina O, Nussenzweig MC (2008). The efficacy of DNA vaccination is enhanced in mice by targeting the encoded protein to dendritic cells. J Clin Invest.

[B12] Barouch DH (2006). Rational design of gene-based vaccines. J Pathol.

[B13] Guimaraes-Walker A, Mackie N, McCormack S, Hanke T, Schmidt C, Gilmour J, Barin B, McMichael A, Weber J, Legg K, Babiker A, Hayes P, Gotch F, Smith C, Dally L, Dorrell L, Cebere I, Kay R, Winstone N, Moore S, Goonetilleke N, Fast P, IAVI-006 Study Group (2008). Lessons from IAVI-006, a Phase I clinical trial to evaluate the safety and immunogenicity of the pTHr.HIVA DNA and MVA.HIVA vaccines in a prime-boost strategy to induce HIV-1 specific T-cell responses in healthy volunteers. Vaccine.

[B14] McCormack S, Stohr W, Barber T, Bart PA, Harari A, Moog C, Ciuffreda D, Cellerai C, Cowen M, Gamboni R (2008). EV02: a Phase I trial to compare the safety and immunogenicity of HIV DNA-C prime-NYVAC-C boost to NYVAC-C alone. Vaccine.

[B15] Harari A, Bart PA, Stohr W, Tapia G, Garcia M, Medjitna-Rais E, Burnet S, Cellerai C, Erlwein O, Barber T (2008). An HIV-1 clade C DNA prime, NYVAC boost vaccine regimen induces reliable, polyfunctional, and long-lasting T cell responses. J Exp Med.

[B16] Tenbusch M, Kuate S, Tippler B, Gerlach N, Schimmer S, Dittmer U, Uberla K (2008). Coexpression of GM-CSF and antigen in DNA prime-adenoviral vector boost immunization enhances polyfunctional CD8+ T cell responses, whereas expression of GM-CSF antigen fusion protein induces autoimmunity. BMC Immunol.

[B17] Kuate S, Stefanou D, Hoffmann D, Wildner O, Uberla K (2004). Production of lentiviral vectors by transient expression of minimal packaging genes from recombinant adenoviruses. J Gene Med.

[B18] Stahl-Hennig C, Kuate S, Franz M, Suh YS, Stoiber H, Sauermann U, Tenner-Racz K, Norley S, Park KS, Sung YC, Steinman R, Racz P, Uberla K (2007). Atraumatic oral spray immunization with replication-deficient viral vector vaccines. J Virol.

[B19] Stone GW, Barzee S, Snarsky V, Kee K, Spina CA, Yu XF, Kornbluth RS (2006). Multimeric soluble CD40 ligand and GITR ligand as adjuvants for human immunodeficiency virus DNA vaccines. J Virol.

[B20] Betts MR, Brenchley JM, Price DA, De Rosa SC, Douek DC, Roederer M, Koup RA (2003). Sensitive and viable identification of antigen-specific CD8+ T cells by a flow cytometric assay for degranulation. J Immunol Methods.

[B21] Robinson HL, Amara RR (2005). T cell vaccines for microbial infections. Nat Med.

[B22] Williams MA, Tyznik AJ, Bevan MJ (2006). Interleukin-2 signals during priming are required for secondary expansion of CD8+ memory T cells. Nature.

[B23] Kanzler H, Barrat FJ, Hessel EM, Coffman RL (2007). Therapeutic targeting of innate immunity with Toll-like receptor agonists and antagonists. Nat Med.

[B24] Andrejeva J, Childs KS, Young DF, Carlos TS, Stock N, Goodbourn S, Randall RE (2004). The V proteins of paramyxoviruses bind the IFN-inducible RNA helicase, mda-5, and inhibit its activation of the IFN-beta promoter. Proc Natl Acad Sci USA.

[B25] Kawai T, Akira S (2007). Antiviral signaling through pattern recognition receptors. J Biochem.

[B26] Seder RA, Darrah PA, Roederer M (2008). T-cell quality in memory and protection: implications for vaccine design. Nat Rev Immunol.

[B27] Barouch DH (2006). Rational design of gene-based vaccines. J Pathol.

[B28] Klempa B, Kruger DH, Auste B, Stanko M, Krawczyk A, Nickel KF, Uberla K, Stang A (2009). A Novel Cardiotropic Murine Adenovirus Representing a Distinct Species of Mastadenoviruses. J Virol.

[B29] Liu J, Kjeken R, Mathiesen I, Barouch DH (2008). Recruitment of antigen-presenting cells to the site of inoculation and augmentation of human immunodeficiency virus type 1 DNA vaccine immunogenicity by in vivo electroporation. J Virol.

[B30] Napolitani G, Rinaldi A, Bertoni F, Sallusto F, Lanzavecchia A (2005). Selected Toll-like receptor agonist combinations synergistically trigger a T helper type 1-polarizing program in dendritic cells. Nat Immunol.

[B31] Zheng R, Cohen PA, Paustian CA, Johnson TD, Lee WT, Shu S, Koski GK (2008). Paired Toll-like receptor agonists enhance vaccine therapy through induction of interleukin-12. Cancer Res.

[B32] Zhu Q, Egelston C, Vivekanandhan A, Uematsu S, Akira S, Klinman DM, Belyakov IM, Berzofsky JA (2008). Toll-like receptor ligands synergize through distinct dendritic cell pathways to induce T cell responses: implications for vaccines. Proc Natl Acad Sci USA.

